# Integrin α5β1 in pancreatic ductal adenocarcinoma: Tumour‒stroma crosstalk, hypoxia and therapeutic targeting

**DOI:** 10.1002/ctm2.70759

**Published:** 2026-08-10

**Authors:** Chenzhe Ma, Yingying Wang, Yumin Li

**Affiliations:** ^1^ The Second Clinical Medical School Lanzhou University Lanzhou China; ^2^ Gansu Provincial Key Laboratory of Environmental Oncology Lanzhou University Second Hospital Lanzhou China

**Keywords:** hypoxia, integrin α5β1, pancreatic ductal adenocarcinoma, volociximab

## Abstract

**Background:**

Pancreatic ductal adenocarcinoma (PDAC) is one of the most lethal malignancies and is characterised by aggressive biological behaviour, marked therapeutic resistance and a dense desmoplastic tumour microenvironment. Integrin α5β1, the principal fibronectin receptor, has emerged as an important mediator of tumour‐stroma interactions through its roles in cell adhesion, mechanotransduction, migration, survival and extracellular matrix (ECM) remodelling.

**Main body:**

Increasing evidence indicates that integrin alpha 5 (ITGA5)/integrin α5β1 is upregulated in PDAC cells and stromal compartments and is associated with invasion, fibrosis, therapeutic resistance and poor prognosis. Hypoxia, a defining feature of PDAC, may further enhance α5β1‐related signalling by promoting stromal activation and ECM remodelling. This review summarises the structural and signalling features of integrin α5β1, its role in hypoxia‐related interactions between tumour cells and the stroma, its contribution to therapeutic resistance in PDAC, and current therapeutic strategies targeting this integrin.

**Conclusion:**

Integrin α5β1 is a biologically relevant therapeutic target for modulating the fibrotic stroma and overcoming treatment resistance in PDAC, and it may also be implicated in tumorstroma crosstalk within the hypoxic microenvironment. Successful clinical translation of α5β1‐targeted strategies will likely require further mechanistic investigation, biomarker‐guided patient selection, and rational combination approaches to address pathway redundancy and the limited efficacy of monotherapy.

## Key points

- Integrin α5β1 drives pancreatic ductal adenocarcinoma tumour‒stroma crosstalk through fibronectin‐dependent adhesion and signalling.
- ITGA5 upregulation is associated with desmoplasia, invasion, chemoresistance and poor prognosis.
- Hypoxia may amplify α5β1‐mediated ECM remodelling and stromal activation.
- Targeting α5β1 may improve chemotherapy response by disrupting stromal barriers.

## Graphical abstract

ITGA5/integrin α5β1 promotes PDAC progression by integrating fibronectin‐dependent adhesion, hypoxia‐associated ECM remodelling, pancreatic stellate cell/cancer‐associated fibroblast activation, desmoplasia and tumour‒stroma crosstalk. These processes contribute to impaired drug delivery and therapeutic resistance. Targeting α5β1 with antibodies or peptide antagonists may alleviate stromal barriers and enhance chemotherapy efficacy.

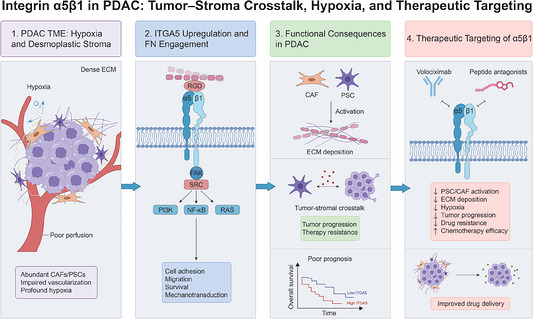

## 1 INTRODUCTION

Pancreatic ductal adenocarcinoma (PDAC) is among the most lethal malignancies and is characterised by an exceptionally poor prognosis. Over the past several decades, the global burden of pancreatic cancer has increased dramatically, with the number of new cases rising from approximately 195 000 in 1990 to 510 992 in 2022.^1^ According to the Global Burden of Disease (GBD) 2023 estimates, the age‐standardised incidence rate (ASIR) of pancreatic cancer in China is 5.1 per 100 000, while the age‐standardised mortality rate (ASMR) reaches 5.0 per 100 000, underscoring its extremely high fatality rate.^2^

Despite advances in surgical techniques, chemotherapy and targeted therapies, therapeutic outcomes for PDAC remain highly unsatisfactory. One of the major obstacles to effective treatment is the unique tumour microenvironment (TME), which is characterised by extensive desmoplastic stroma and sparse, poorly functional microvasculature. This distinctive architecture generates a formidable physical and biological barrier that severely limits drug delivery and contributes to therapeutic resistance.^3^ The stroma of PDAC is mainly composed of cancer‐associated fibroblasts (CAFs), immune cells, vascular components and dense extracellular matrix (ECM). These stromal elements interact with tumour cells in a highly dynamic and reciprocal manner. CAFs serve as the dominant functional cell population within the stroma, producing large amounts of ECM components such as collagen, fibronectin (FN) and laminin, and releasing diverse cytokines and growth factors. Together, these stromal processes support pancreatic tumour growth, angiogenesis, invasion and metastasis.^4^ Moreover, impaired vascular perfusion, together with excessive ECM deposition, generates a state of profound and sustained intratumoural hypoxia, which critically contributes to the invasive phenotype of PDAC.^5^

Hypoxia‐inducible factor‐1 (HIF‐1) serves as a master regulator of cellular adaptation to hypoxic stress and plays a central role in PDAC progression.^6^ Under hypoxic conditions, stabilisation of HIF‐1α activates HIF‐1‐dependent transcriptional programs that drive metabolic reprogramming, angiogenic factor production, epithelial‒mesenchymal transition (EMT) and stromal remodelling. Importantly, emerging evidence suggests that hypoxia and HIF‐1α signalling critically regulate tumour‒stroma interactions by modulating the expression of adhesion molecules and ECM receptors, thereby reinforcing the aggressive phenotype of PDAC.^7^

Integrin α5, encoded by the integrin alpha 5 (ITGA5) gene, noncovalently associates with integrin β1 (ITGB1) to form the α5β1 heterodimer, a principal receptor for FN.^8^ The extracellular domain of integrin α5β1 recognises arginine‒glycine‒aspartate (RGD) motifs within FN and fibrinogen, enabling cell‒ECM adhesion and mechanotransduction.^9^, ^10^, ^11^ Through activation of downstream signalling pathways, integrin α5β1 plays a critical role in regulating cell adhesion, migration, survival and signal transduction, and has been implicated in tumour invasion, metastasis and resistance to chemotherapy.^12^, ^13^, ^14^

Given the central roles of desmoplasia, hypoxia and tumour‒stroma crosstalk in PDAC progression, integrin α5β1 has emerged as an important mediator linking ECM remodelling to therapeutic resistance. In this review, we summarise current advances in understanding the biological functions and molecular mechanisms of integrin α5β1 in pancreatic cancer, with particular emphasis on its role in ECM remodelling and treatment resistance within the hypoxic TME. Furthermore, we discuss the therapeutic potential of targeting integrin α5β1 to alleviate desmoplasia, improve drug delivery and treatment sensitivity, and enhance clinical outcomes in patients with PDAC.

## 2 STRUCTURAL FEATURES AND BIDIRECTIONAL SIGNALLING OF INTEGRIN α5β1

**FIGURE 1:**
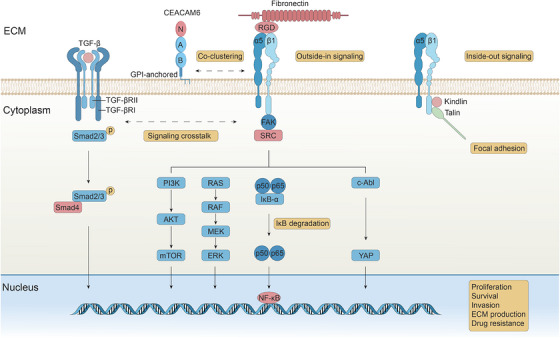
Schematic overview of integrin α5β1‐centred signalling in pancreatic ductal adenocarcinoma (PDAC). Transforming growth factor‐β (TGF‐β) activates canonical Smad2/3 signalling and functionally crosstalks with fibronectin (FN)‒α5β1/focal adhesion kinase (FAK) signalling during pancreatic stellate cell (PSC)/cancer‐associated fibroblast (CAF) activation. FN binding to α5β1 triggers outside‐in signalling through FAK/SRC and downstream phosphoinositide‐3‐kinase (PI3K)‒protein kinase B (AKT)‒mechanistic target of rapamycin (mTOR), rat sarcoma (RAS)‒extracellular signal‐regulated kinase (ERK), nuclear factor kappa‐light‐chain‐enhancer of activated B cells (NF‐κB) and c‐Abl/YAP pathways. Conversely, talin/kindlin‐mediated inside‐out signalling promotes α5β1 activation and focal adhesion assembly. CEACAM6 may enhance FN‒α5β1 engagement through membrane co‐clustering.

The integrin family consists of 18 α subunits and eight β subunits that assemble into 24 distinct heterodimeric transmembrane adhesion receptors. These receptors are capable of transducing both physical and biochemical signals from the ECM into intracellular signalling cascades, thereby playing central roles in tumour cell adhesion, migration, invasion, angiogenesis and therapeutic resistance.^15^, ^16^, ^17^ Both α and β subunits share a conserved structural organisation, comprising a large extracellular domain, a single‐pass transmembrane domain, and a relatively short cytoplasmic tail.^18^ The extracellular domains function as sensory interfaces that enable cells to perceive and respond to microenvironmental cues, including adhesive ligands and growth factors.^19^ In contrast, the cytoplasmic tails connect integrins to the actin cytoskeleton and intracellular signalling pathways (Figure 1), such as Src family kinases, focal adhesion kinase (FAK), mitogen‐activated protein kinase (MAPK) and protein kinase B (AKT), thereby coordinating cytoskeletal dynamics with signal transduction.^20^, ^21^, ^22^, ^23^

### 2.1 Integrin α5β1 ligand recognition and molecular interactions

Integrin α5β1 is a prototypical receptor for FN, exhibiting high specificity for extracellular ligands that contain the RGD tripeptide motif.^24^ This RGD sequence, which is conserved across numerous ECM proteins, functions as a critical recognition site mediating stable cell‒matrix adhesion. Among RGD‐containing ligands, FN represents the primary and most extensively studied binding partner of integrin α5β1, acting as a central mediator of adhesion, mechanotransduction and intracellular signalling.^25^, ^26^, ^27^, ^28^, ^29^, ^30^, ^31^

FN is a large multidomain glycoprotein that is abundantly deposited within the tumour stroma, particularly in desmoplastic malignancies such as PDAC, where it contributes to the formation of a dense and mechanically active ECM.^32^, ^33^ Engagement of α5β1 with FN not only anchors cells to the ECM but also induces integrin clustering, focal adhesion assembly and activation of downstream signalling pathways that regulate cellular proliferation, migration and invasive behaviour.^34^, ^35^

In addition to FN, several other molecules have been reported to interact with integrin α5β1. Vascular endothelial growth factor receptor‐1 (VEGFR‐1) has been shown to be deposited within the ECM by endothelial cells, where it functions as a ligand for integrin α5β1. Interaction between matrix‐associated VEGFR‐1 and integrin α5β1 enhances endothelial cell adhesion and migration, thereby facilitating angiogenic processes.^36^, ^37^ Cell surface or soluble molecules, such as CD97, uPAR/CD87 and CD154/CD40L, have also been implicated in α5β1‐dependent adhesion and signalling, contributing to cell migration, inflammatory responses and angiogenesis.^38^, ^39^, ^40^ Moreover, neuropilin‐2,^41^ tubulointerstitial nephritis antigen‐like 1 (Tinagl1),^42^ pregnancy‐specific glycoprotein 1 (PSG1),^43^ porcine haemagglutinating encephalomyelitis virus (PHEV)^44^ and 25‐hydroxycholesterol^45^ have also been identified as α5β1‐binding partners, further expanding the functional spectrum of this integrin in cell‒matrix communication and signal transduction.

### 2.2 Bidirectional signalling and regulatory mechanisms of integrin α5β1

Integrin α5β1 exhibits the characteristic bidirectional signalling properties shared by integrin family members.^46^ On the one hand, engagement of ECM ligands such as FN initiates ‘outside‐in’ signalling, leading to the assembly of focal adhesion complexes and activation of downstream pathways. Through these signalling networks, integrin α5β1 regulates cytoskeletal remodelling, cell migration, proliferation and survival, thereby contributing to tumour progression and therapeutic resistance.^23^ On the other hand, intracellular signalling events can induce conformational changes in integrin α5β1 via ‘inside‐out’ signalling mechanisms (Figure 1). This process enhances ligand‐binding affinity and avidity by promoting integrin activation and clustering at the cell surface, thereby strengthening cell‒ECM adhesion and generating a positive feedback loop that amplifies integrin‐mediated signalling.

Beyond canonical bidirectional signalling, the expression and functional activity of ITGA5 are subject to multilayered regulation. Post‐transcriptional regulation by microRNAs, post‐translational modifications such as glycosylation, and tightly controlled integrin trafficking processes, including endocytosis, recycling and membrane redistribution, collectively fine‐tune integrin α5β1 availability and signalling output.^37^, ^47^, ^48^, ^49^, ^50^ These regulatory mechanisms contribute to the context‐dependent and heterogeneous roles of integrin α5β1 across different tumour types and cellular states. Overall, the dynamic bidirectional signalling capacity of integrin α5β1 enables cells to continuously sense, interpret and adapt to changes in the TME, thereby orchestrating essential biological processes such as adhesion, migration, proliferation and survival.

## 3 ONCOGENIC ROLES OF INTEGRIN α5β1 IN PANCREATIC CANCER

### 3.1 Upregulation and clinical relevance of ITGA5 in PDAC

Accumulating evidence indicates that integrin α5β1 is aberrantly expressed in PDAC and is associated with tumour progression and poor clinical outcomes. Increased expression of ITGA5 has been detected in pancreatic cancer tissues compared with normal pancreatic epithelium and is frequently correlated with aggressive tumour behaviour, including enhanced invasiveness and metastatic potential.^51^, ^52^ Clinical studies have further demonstrated that elevated ITGA5 expression is associated with reduced overall survival in patients with PDAC.^53^ In addition to tumour cells, integrin α5β1 is also highly expressed in stromal components such as CAFs and pancreatic stellate cells (PSCs), highlighting its important role in shaping the desmoplastic TME.^51^, ^54^

### 3.2 Loss‐of‐function evidence supporting the target specificity of ITGA5 in PDAC

Beyond correlative expression evidence, loss‐of‐function studies further support the functional specificity and therapeutic relevance of ITGA5 in PDAC. In MiaPaCa‐2 pancreatic carcinoma cells, Lowrie et al.^55^ demonstrated that antibody‐mediated inhibition of α5 integrin suppressed FN‐induced cell spreading and proliferation, supporting a functional role for α5β1‐containing adhesion complexes in tumour cell‒ECM signalling. In PSCs, Kuninty et al.^51^ demonstrated that ITGA5 knockdown inhibited human PSC differentiation, whereas pharmacological targeting with AV3‐attenuated stromal activation and enhanced gemcitabine efficacy in preclinical PDAC models. Consistently, Wang et al.^54^ showed that ITGA5 blockade in PSCs using AV3 peptide or siRNA impaired PSC proliferation and attenuated tumour‒stromal crosstalk in vitro. Notably, silencing ITGA5 in PSCs largely abolished PSC‐mediated enhancement of drug resistance, migration, invasion and cancer stem cell‐like phenotypes in SW1990 cells. Although complete ITGA5 knockout models in PDAC remain limited, available antibody‐blocking, knockdown, siRNA‐mediated inhibition and pharmacological blockade studies collectively support a specific functional role of ITGA5 in tumour cell‒ECM signalling, PSC activation, stromal remodelling and therapeutic relevance.

### 3.3 Potential functional convergence between CEACAM6 and integrin α5β1 in PDAC

CEACAM6, also known as CD66c or nonspecific cross‐reacting antigen, is a glycosylphosphatidylinositol‐anchored cell surface glycoprotein belonging to the carcinoembryonic antigen‐related cell adhesion molecule (CEACAM) family.^56^ In PDAC, CEACAM6 is frequently upregulated in PDAC tissues and pancreatic intraepithelial neoplasia (PanIN) lesions, and its expression has been associated with lymph node metastasis, advanced disease stage, poor postoperative survival and chemoresistance.^57^, ^58^ Functional studies further indicate that CEACAM6 contributes to pancreatic cancer aggressiveness. Its overexpression enhances cellular invasiveness through c‐Src‐dependent regulation of matrix metalloproteinase‐9 (MMP‐9) activity, whereas its knockdown attenuates invasion.^59^ In addition, CEACAM6 has been shown to promote EMT, migration, invasion and metastasis in pancreatic cancer, partly through ZEB1/ZEB2‐associated signalling.^60^ Integrative analyses further suggest that CEACAM6 affects ECM‒cell adhesion, fibrotic reaction, immune regulation and tumour metabolism in PDAC.^61^

Although direct physical binding between CEACAM6 and integrin α5β1 has not been demonstrated in PDAC, available evidence supports a functional relationship between CEACAM6 and integrin‐mediated adhesion signalling. In other cellular systems, CEA and CEACAM6 have been shown to co‐cluster with integrin α5β1 at the cell surface and enhance its interaction with FN.^62^, ^63^ This functional coupling promotes cell adhesion to FN, FN matrix assembly and resistance to anoikis, suggesting that CEACAM6 may regulate α5β1 activity through membrane organisation. In BxPC‐3 cells, CEACAM6 crosslinking was shown to enhance adhesion to ECM components through c‐Src‐dependent functional crosstalk with integrin αvβ3, demonstrating that CEACAM6 can engage integrin‐associated signalling in pancreatic cancer cells.^64^ Whether a comparable functional coupling with integrin α5β1 occurs in PDAC remains unclear. Further biochemical and proximity‐based studies are required to determine whether CEACAM6 and integrin α5β1 are physically associated or functionally coordinated within shared membrane signalling complexes in PDAC.

### 3.4 Cell‐type‐specific roles of integrin α5β1 in PDAC

**FIGURE 2:**
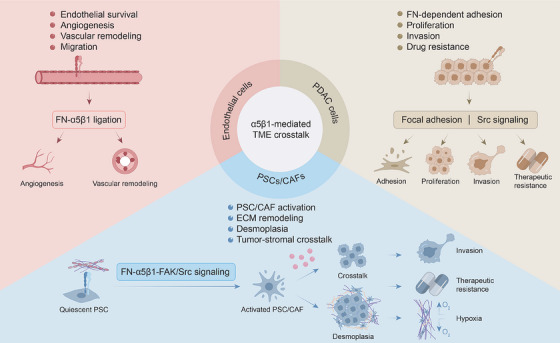
Cell‐type‐specific roles of integrin α5β1 in pancreatic ductal adenocarcinoma (PDAC). Integrin α5β1 exerts distinct functions across different cellular compartments within the PDAC tumour microenvironment. In tumour cells, α5β1 promotes fibronectin (FN)‐dependent adhesion, focal adhesion/Src signalling, proliferation, invasion and therapy resistance. In pancreatic stellate cells (PSCs)/cancer‐associated fibroblasts (CAFs), α5β1 signalling drives fibroblast activation, extracellular matrix remodelling, desmoplasia and tumour‒stromal interactions, promoting tumour cell invasion, hypoxia and therapeutic resistance. In endothelial cells, FN‒α5β1 signalling regulates endothelial migration, angiogenesis and vascular remodelling.

The biological effects of integrin α5β1 in PDAC are highly dependent on the cellular context (Figure 2). In PDAC cells, integrin α5β1 primarily promotes FN‐dependent adhesion, migration and invasion by regulating integrin trafficking, focal adhesion turnover and downstream Src‐associated signalling.^65^, ^66^ In PSCs and CAFs, ITGA5 has a more clearly established role in fibroblast activation, ECM production, desmoplasia and paracrine support of tumour cells. Genetic or pharmacological inhibition of stromal ITGA5 reduces PSC activation and migration, attenuates ECM deposition, improves tumour perfusion and enhances the response to gemcitabine in preclinical PDAC models.^51^

In endothelial cells, integrin α5β1 contributes to FN‐dependent adhesion, survival and angiogenesis. Integrin α5β1 and FN are coordinately upregulated in tumour‐associated blood vessels, and ligation of α5β1 by the central cell‐binding domain of FN promotes angiogenesis in vivo.^67^ Mechanistically, ligated integrin α5β1 supports endothelial cell survival by suppressing protein kinase A activity, whereas α5β1 antagonism activates a protein kinase A‐dependent caspase‐8 apoptotic pathway and inhibits angiogenesis.^68^ Beyond its role in endothelial survival and angiogenesis, integrin α5β1 also participates in flow‐responsive mechanotransduction. Under oscillatory shear stress, annexin A2 associates with integrin α5 and facilitates its translocation to lipid rafts, thereby promoting α5β1 activation and inflammatory endothelial responses.^69^ However, these findings were derived mainly from vascular and non‐PDAC models, and their specific relevance to PDAC vasculature remains to be established.

These observations indicate that the contribution of integrin α5β1 to PDAC progression is cell type dependent rather than uniform across the TME. The expression patterns, major functions, downstream pathways and hypoxia/ECM‐related relevance across different cellular compartments are summarised in Table 1.

**TABLE 1:** Cell‐type‐specific roles and hypoxia‐related relevance of integrin α5β1 in pancreatic ductal adenocarcinoma (PDAC).

Cell type	Functions	Mechanisms	Hypoxia/ECM relevance	References
PDAC cells	FN‐dependent adhesion Proliferation Invasive motility	RhoC‐dependent α5β1 trafficking Focal adhesion dynamics Src signalling	Direct hypoxic regulation unconfirmed in PDAC	^55^, ^65^, ^66^
PSCs/CAFs	Fibroblast activation ECM remodelling Tumour‒stromal crosstalk	FN‒α5β1 signalling FAK/Src activation Crosstalk with TGF‐β/Smad signalling	Hypoxia‐induced PSC activation Increased FN and collagen production Indirect enhancement of α5β1 signalling	^51^, ^54^, ^70^
Endothelial cells	Endothelial survival Angiogenesis	FN‒α5β1 ligation PKA suppression	HIF‐1α‐associated angiogenesis α5β1 role unconfirmed in PDAC	^67^, ^68^, ^71^

Abbreviations: CAF, cancer‐associated fibroblast; ECM, extracellular matrix; FAK, focal adhesion kinase; FN, fibronectin; HIF‐1α, hypoxia‐inducible factor‐1; PKA, protein kinase A; PSC, pancreatic stellate cell; TGF‐β, transforming growth factor‐β.

### 3.5 Integrin α5β1‐mediated signalling pathways in PDAC

#### 3.5.1 Integrin α5β1‒FAK/Src signalling pathway

Integrin α5β1 contributes to pancreatic cancer cell proliferation and survival through the activation of multiple intracellular signalling pathways. FAK/Src signalling pathway represents the most extensively characterised downstream signalling cascade of integrin α5β1 in PDAC.^72^, ^73^, ^74^, ^75^ Upon binding to FN in the ECM, integrin α5β1 undergoes conformational activation and clustering at the cell membrane, leading to recruitment and phosphorylation of FAK. Activated FAK then interacts with Src family kinases to form a focal adhesion signalling complex, orchestrating cytoskeletal remodelling, focal adhesion turnover and directional migration.^52^ This signalling hub underlies tumour cell adhesion, motility, invasion and survival, providing a mechanistic basis for PDAC progression and resistance to therapy.^76^

Within the pancreatic TME, integrin α5β1 signalling also cooperates with transforming growth factor‐β (TGF‐β) pathways, particularly in PSCs, which are the principal drivers of stromal fibrosis in PDAC. As a pleiotropic cytokine, TGF‐β promotes tumour progression by suppressing antitumour immunity, enhancing angiogenesis and regulating both cancer cells and the TME through autocrine and paracrine signalling.^77^, ^78^ Engagement of FN by integrin α5β1 activates the integrin α5β1/FN‒FAK‒Src signalling axis, which functionally synergises with TGF‐β/Smad2 signalling to promote PSC activation and ECM production.^51^, ^79^ This coordinated signalling network establishes a dense, fibrotic TME that reinforces tumour‒stroma crosstalk and promotes a supportive niche for cancer cell proliferation, invasion and therapy resistance.

In addition to regulating cytoskeletal and stromal signalling, integrin‐mediated adhesion has also been shown to modulate inflammatory signalling pathways in pancreatic cancer cells. In FG pancreatic cancer cells, integrin‐mediated signalling may be associated with protein kinase B (PKB)/Akt and GSK3 activity, leading to increased expression of inflammatory mediators such as parathyroid hormone‐related protein (PTHrP), interleukin‐6 (IL‐6) and interleukin‐8 (IL‐8).^80^ These inflammatory mediators are known to promote tumour progression and tumour‒stroma interactions, suggesting that integrin‐dependent signalling may further contribute to PDAC development by regulating inflammatory and paracrine communication within the TME.

#### 3.5.2 Integrin α5β1‒phosphoinositide‐3‐kinase/AKT signalling pathway

In addition to the FAK/Src signalling axis, integrin α5β1 engagement with ECM components can activate the phosphoinositide‐3‐kinase (PI3K)/AKT signalling pathway, which plays a critical role in cell survival, growth and cell cycle progression.^81^ Although direct evidence linking ITGA5 to PI3K/AKT activation in PDAC remains relatively limited compared with the well‐established FAK pathway, studies in PSCs have demonstrated that interactions between ECM components and β1 integrins can stimulate AKT phosphorylation and promote cell proliferation.

Upon engagement with FN, integrin α5β1 undergoes conformational activation and clustering at the cell surface, facilitating recruitment and activation of FAK. Activated FAK subsequently associates with PI3K, triggering AKT phosphorylation and downstream signalling events that promote cyclin D1 expression and cell proliferation. Through this mechanism, integrin α5β1 effectively transduces ECM‐derived cues into intracellular survival and growth signalling.^82^

Importantly, accumulating evidence from other malignancies further supports a functional connection between ITGA5 and PI3K/AKT signalling. For example, ITGA5 has been shown to promote tumour proliferation and invasion through activation of the FAK/PI3K/AKT signalling pathway in gastric cancer, colorectal cancer and glioma,^83^, ^84^, ^85^ while similar signalling axes have been implicated in drug resistance and metastatic behaviour in lung cancer.^86^

#### 3.5.3 Integrin α5β1‒MAPK/ERK signalling pathway

MAPK/ERK (extracellular signal‐regulated kinase) pathway represents another downstream signalling cascade that can be activated by integrin‐mediated adhesion.^87^ Upon engagement with ECM ligands, integrin signalling may stimulate the rat sarcoma‒rapidly accelerated fibrosarcoma‒mitogen‐activated protein kinase kinase‒extracellular signal‐regulated kinase (RAS‒RAF‒MEK‒ERK) cascade, leading to transcriptional programs that regulate cell proliferation, differentiation and migration.^82^ Although direct evidence specifically linking ITGA5 to MAPK/ERK activation in PDAC remains relatively limited compared with the FAK pathway, several studies have demonstrated that integrin β1‐mediated interactions with the ECM can stimulate ERK phosphorylation in PSCs.^88^ Since activated PSCs are the primary source of ECM in PDAC, integrin‐dependent MAPK activation may indirectly promote tumour progression by enhancing stromal remodelling and tumour‒stroma crosstalk within the pancreatic TME.

#### 3.5.4 Integrin α5β1‒NF‐κB signalling pathway

Nuclear factor kappa‐light‐chain‐enhancer of activated B cells (NF‐κB) is a central mediator of inflammation‐associated tumourigenesis and regulates diverse transcriptional programs involved in cell survival, proliferation, metastasis, angiogenesis and therapeutic resistance.^89^ Accumulating evidence indicates that integrin α5β1 is functionally linked to NF‐κB signalling across multiple cellular contexts. In human bronchial epithelial cells, FN promotes cell proliferation and inhibits apoptosis through α5β1‐dependent activation of the PI3K/Akt pathway, which in turn enhances NF‐κB signalling. This activation leads to increased expression of c‐Myc and cyclin D1, accompanied by downregulation of p21 and PTEN, thereby driving pro‐survival and pro‐proliferative transcriptional programs.^90^ Consistent with this, lunasin, a peptide that directly binds integrin α5β1, suppresses FAK/ERK/NF‐κB signalling in colon cancer cells. Mechanistically, lunasin upregulates inhibitor of nuclear factor kappa B alpha (IκB‐α) and reduces nuclear NF‐κB p50 levels, indicating that inhibition of α5β1‐associated signalling attenuates NF‐κB activation and impairs metastatic potential.^91^ Together, these findings identify NF‐κB as a key downstream effector of α5β1 signalling in the regulation of proliferation, survival and metastatic progression. Additional support comes from endothelial cell studies showing that fibrinogen‐induced NF‐κB activation and chemokine expression can be inhibited by antibodies targeting α5β1 and αvβ3 integrins. Although this does not establish a tumour cell‐intrinsic mechanism specific to ITGA5, it highlights a broader role for α5β1 in mediating ECM‐driven inflammatory signalling within the TME.^92^

#### 3.5.5 Integrin α5β1‒YAP signalling pathway

The YAP pathway is increasingly recognised as a critical integrator of mechanical and microenvironmental cues in cancer.^93^ Emerging evidence indicates that integrin α5β1 may function as an upstream mechanosensory regulator of YAP signalling. In endothelial cells under disturbed flow, FN‐dependent α5β1 activation enhances c‐Abl‐mediated YAP Tyr357 phosphorylation and the expression of proinflammatory genes.^94^ In Ewing sarcoma, tenascin C promotes tumour progression through integrin α5β1‐mediated YAP activation and MALAT1 upregulation.^95^ In atherosclerosis‐related models, α5β1 regulates YAP through a PDE4D/PP2A‐dependent pathway, suggesting that α5β1 may influence YAP activity through distinct upstream mechanisms. Furthermore, in pancreatic progenitors, a FN integrin α5β1 YAP1 axis has been identified as a key mechanotransduction pathway controlling cell fate decisions.^96^ Additional tumour evidence further suggests that the relationship between α5β1 and YAP can be bidirectional. In breast cancer, serglycin (SRGN) sustains chemoresistance and stemness partly through an ITGA5/FAK/CREB‐dependent increase in YAP transcription, supporting a positive regulatory loop between ITGA5‐associated signalling and YAP activity.^97^ In ovarian cancer, PPP2CA‐related dysregulation has been linked to enhanced YAP nuclear translocation together with increased ITGA5 expression, implying that YAP activation may in turn reinforce integrin‐dependent malignant programs.^98^

## 4 INTEGRIN α5β1 IN THE HYPOXIC TME

HIF‐1 is a heterodimeric transcription factor composed of an oxygen‐regulated α subunit, HIF‐1α, and a constitutively expressed β subunit, HIF‐1β, also known as aryl hydrocarbon receptor nuclear translocator (ARNT). The HIF‐α family includes HIF‐1α, HIF‐2α and HIF‐3α, which can heterodimerise with HIF‐1β/ARNT to regulate hypoxia‐responsive transcriptional programs.^99^ PDAC is characterised by a profoundly hypoxic TME due to aberrant vascularisation, poor perfusion and extensive desmoplastic stroma.^100^ Hypoxia drives tumour progression primarily through the stabilisation and activation of HIFs, which regulate genes involved in angiogenesis, metabolic adaptation, ECM remodelling and cell survival. The hypoxic niche also promotes EMT, enhances migratory and invasive capacities, and fosters cancer stem‐like cell phenotypes, thereby collectively contributing to aggressive tumour behaviour.^5^, ^101^

PDAC‐specific evidence directly supports a close association between hypoxia and stromal ECM remodelling. Hypoxia enhances PSC activity and increases the production of FN, type I collagen, periostin and VEGF, thereby promoting a fibrotic microenvironment and reinforcing the hypoxia‒fibrosis cycle.^70^ These stromal changes may create an ECM context that favours FN/integrin α5β1‐mediated adhesion and signalling.

Mechanistic evidence directly linking hypoxia to integrin α5β1 has been reported in other tumour types. For instance, hypoxia selectively enhances integrin α5β1 expression in breast cancer cells, thereby promoting metastatic dissemination.^102^ Similarly, HIF‐1α can directly upregulate integrin α5 and FN expression in oral squamous cell carcinoma, facilitating tumour invasion and matrix‐dependent signalling.^103^ In addition to direct transcriptional regulation, hypoxia‐induced HIF‐1α signalling has also been shown to enhance integrin α5β1 activity indirectly. In head and neck squamous cell carcinoma, HIF‐1α upregulates Tensin‐4 (TNS4), which stabilises the integrin α5β1 complex and promotes activation of FAK‐dependent AKT and TGF‐β signalling pathways.^104^ These studies demonstrate that hypoxia can regulate the expression, stability or activity of integrin α5β1, but whether comparable mechanisms operate in PDAC remains to be determined.

**FIGURE 3:**
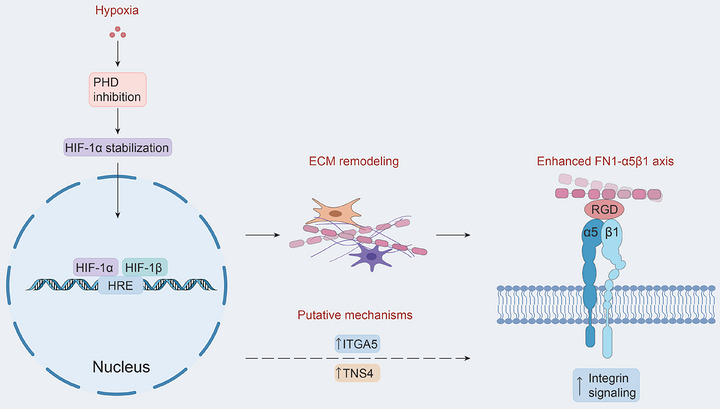
Proposed mechanisms linking hypoxia to fibronectin (FN)/integrin α5β1 signalling in pancreatic ductal adenocarcinoma (PDAC). Hypoxia may enhance integrin α5β1 signalling indirectly through stromal activation and extracellular matrix (ECM) remodelling, or through putative direct mechanisms involving increased ITGA5 expression and stabilisation of integrin α5β1 by Tensin‐4 (TNS4).

Taken together, hypoxia may enhance integrin α5β1 signalling in PDAC through both direct and indirect mechanisms (Figure 3). Based on findings from other tumour types, HIF‐dependent regulation of ITGA5 expression or integrin α5β1 activity is biologically plausible, although it has not yet been experimentally demonstrated in PDAC. Current evidence in PDAC more strongly supports an indirect route in which hypoxia activates PSCs and CAFs and promotes ECM remodelling with increased FN and collagen deposition, thereby creating a dense, fibrotic microenvironment that favours integrin α5β1‐mediated adhesion, mechanotransduction and downstream signalling.

## 5 ROLE OF INTEGRIN α5β1 IN THERAPEUTIC RESISTANCE

**FIGURE 4:**
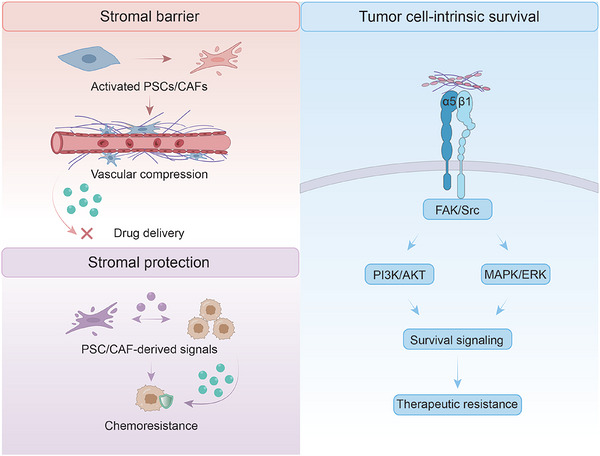
Mechanisms of integrin α5β1‐mediated therapeutic resistance in pancreatic ductal adenocarcinoma (PDAC). α5β1 promotes resistance through stromal barrier formation, pancreatic stellate cell (PSC)/cancer‐associated fibroblast (CAF)‐mediated tumour protection, and tumour cell‐intrinsic survival signalling. These mechanisms collectively impair therapeutic response by reducing drug delivery and enhancing tumour cell survival.

Integrin α5β1 may contribute to therapeutic resistance through both microenvironmental and tumour cell‐intrinsic mechanisms.^105^ These effects can be broadly categorised into impaired drug delivery caused by the fibrotic stroma, PSC/CAF‐mediated protection of tumour cells, and integrin‐dependent survival signalling (Figure 4).

### 5.1 ECM barrier and impaired drug delivery

The dense desmoplastic stroma of PDAC represents a major physical barrier to effective drug delivery. Stromal ITGA5 is highly expressed in PSCs, and its inhibition attenuates desmoplasia, decompresses tumour vasculature, improves perfusion and enhances the antitumour efficacy of gemcitabine in preclinical models, indicating that α5β1 promotes chemoresistance at least in part by reinforcing the fibrotic stromal barrier and limiting drug delivery.^51^

### 5.2 PSC/CAF‐mediated resistance

In addition to forming a physical barrier, PSCs and CAFs can protect pancreatic cancer cells through tumour‒stromal signalling. In vitro inhibition of ITGA5 in PSCs reduces their proliferation and migration and weakens their ability to promote drug resistance, migration, invasion and cancer stem cell‐like phenotypes in pancreatic cancer cells.^54^ These findings suggest that stromal integrin α5β1 also contributes to therapeutic resistance by maintaining tumour‐supportive communication between PSCs and cancer cells.

### 5.3 Tumour cell‐intrinsic survival signalling

Integrin‐mediated adhesion can also promote treatment resistance through tumour cell‐intrinsic survival signalling. β1 integrin‒ECM interactions promote radiotherapy resistance through DNA repair and pro‐survival signalling mediated by FAK, Src, PI3K/AKT and MAPK.^10^, ^106^, ^107^

Evidence from other malignancies further supports a broader role for integrin α5β1 in therapeutic resistance. In epithelial ovarian carcinoma, integrin α5 has been identified as an independent risk factor for chemoresistance and poor prognosis.^108^ Similarly, in K562 chronic myelogenous leukemia cells, adhesion to FN through α5β1 integrin confers resistance to apoptosis induced by breakpoint cluster region‒Abelson fusion oncogene (BCR/ABL) inhibition, cytotoxic drugs and γ‐irradiation.^109^ In glioblastoma, selective α5β1 antagonists reduce chemotherapy‐induced premature senescence and facilitate apoptosis, indicating that α5β1 can blunt cytotoxic responses and alter treatment sensitivity in a p53‐dependent context.^110^ Moreover, in breast cancer, overexpression of integrin α5β1 promotes resistance to doxorubicin through downregulation of ERK.^111^

## 6 THERAPEUTIC TARGETING OF INTEGRIN α5β1

**FIGURE 5:**
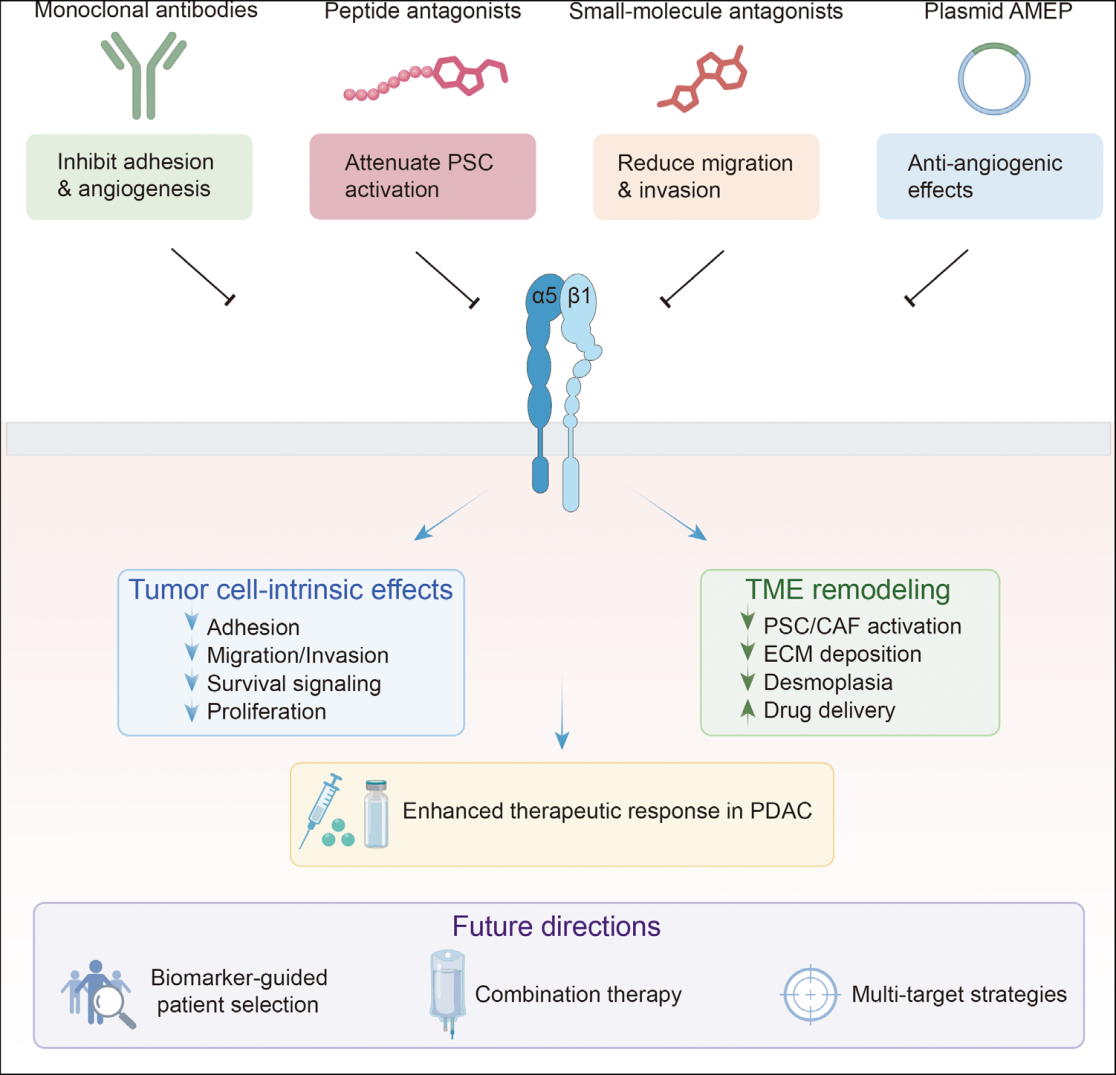
Therapeutic targeting of integrin α5β1 in pancreatic ductal adenocarcinoma (PDAC). Schematic overview of α5β1‐targeting strategies, including monoclonal antibodies, peptide antagonists, small‐molecule antagonists and gene‐based approaches. Inhibition of α5β1 suppresses tumour cell‐intrinsic functions and remodels the tumour microenvironment, leading to enhanced therapeutic responses. Future directions include biomarker‐guided selection, combination therapies and multi‐target strategies.

Given the pivotal role of integrin α5β1 in mediating tumour cell adhesion, migration, survival and tumour‒stroma interactions, targeting this integrin has emerged as a promising therapeutic strategy in cancer^106^ (Figure 5). Integrin α5β1 primarily functions as the major receptor for FN in the ECM, and its activation triggers downstream signalling pathways such as FAK, PI3K/AKT and MAPK/ERK, which collectively promote tumour progression, invasion and resistance to therapy. In PDAC, the FN‒α5β1 axis is closely linked to the desmoplastic TME, where PSCs produce abundant ECM components that reinforce integrin‐mediated signalling. Therefore, targeting integrin α5β1 may simultaneously inhibit tumour cell‐intrinsic signalling and disrupt tumour‒stroma crosstalk, providing a dual therapeutic advantage. An overview of representative α5β1‐targeted agents and related cancer clinical trials is provided in Table 2.

**TABLE 2:** Summary of integrin α5β1‐targeted agents evaluated in cancer clinical trials registered at ClinicalTrials.gov.

Clinical trial	Phase	Drug	Participants	Cancer	Treatment	Locations	Clinical outcomes
NCT00666692	Phase 1	Volociximab (M200)	7	Non‐small‐cell lung cancer	Combination with carboplatin, paclitaxel and bevacizumab	United States	Safety study
NCT00654758	Phase 1	Volociximab (M200)	33	Non‐small‐cell lung cancer	Combination with carboplatin and paclitaxel	NA	Safety study
NCT00635193	Phase 1/2	Volociximab (M200)	138	Ovarian cancer, primary peritoneal cancer	Combination with liposomal doxorubicin	United States	No significant PFS benefit
NCT00516841	Phase 2	Volociximab (M200)	16	Ovarian cancer Peritoneal neoplasms	Monotherapy	United States	No objective response
NCT00401570	Phase 2	Volociximab (M200)	40	Pancreatic cancer	Combination with gemcitabine	United States	Limited activity
NCT00369395	Phase 2	Volociximab (M200)	19	Melanoma	Monotherapy	United States	Insufficient activity
NCT00100685	Phase 2	Volociximab (M200)	48	Renal cell carcinoma Metastases	Monotherapy	United States	Limited activity
NCT00099970	Phase 2	Volociximab (M200)	40	Melanoma Metastases	Combination with dacarbazine	United States	Limited activity
NCT00352313	Phase 1/2	ATN‐161	82	Glioma	Combination with carboplatin	United States	Safety/dose‐finding study
NCT00131651	Phase 2	ATN‐161	36	Renal cell cancer	Monotherapy	United States	Dose‐finding study
NCT01664273	Phase 1	Plasmid AMEP	7	Metastatic malignant neoplasm	Monotherapy	Denmark	Safety/dose‐finding study
NCT00915278	Phase 1	PF‐04605412	33	Non‐haematologic malignancies	Monotherapy	United States	No antitumour activity

Abbreviation: PFS, progression‐free survival.

### 6.1 Monoclonal antibodies

#### 6.1.1 Volociximab

One of the earliest therapeutic approaches developed to inhibit α5β1 signalling involved monoclonal antibodies targeting this integrin. Volociximab (M200), a chimeric monoclonal antibody against integrin α5β1, was designed to block the interaction between α5β1 and FN, thereby inhibiting integrin‐mediated cell adhesion and angiogenesis.^112^, ^113^ Preclinical studies demonstrated that volociximab suppresses endothelial cell migration and tumour angiogenesis, leading to reduced tumour growth in several cancer models.^114^, ^115^ In breast cancer bone metastasis models, pharmacological blockade of α5β1 with volociximab significantly reduced tumour cell adhesion to FN, inhibited tumour cell colonisation of the bone marrow, and delayed the development of skeletal tumour burden and osteolytic lesions.^116^ These findings further support the therapeutic value of α5β1 inhibition in limiting tumour dissemination and tumour‐associated bone destruction.

Early clinical studies showed that volociximab could be administered to patients with advanced solid tumours and achieve sustained occupancy of α5β1 binding sites on circulating cells.^117^ Importantly, volociximab was evaluated in combination with gemcitabine in a multicentre, open‐label phase II trial involving previously untreated patients with metastatic pancreatic cancer (NCT00401570). Among the 20 patients in the reported cohort, one achieved a partial response and 10 had stable disease, with a median overall survival of 5.4 months.^118^ Although the combination was considered feasible, the low objective response rate and single‐arm design precluded a clear assessment of the additional clinical benefit of volociximab. Interpretation was further limited by the small cohort size and lack of biomarker‐guided patient selection.

Clinical studies in other malignancies similarly demonstrated limited or context‐dependent activity. In platinum‐resistant epithelial ovarian cancer, volociximab monotherapy was generally well tolerated but demonstrated limited antitumour efficacy.^119^ In advanced non‐small‐cell lung cancer (NSCLC), its combination with carboplatin and paclitaxel showed manageable toxicity and preliminary clinical activity.^120^ Overall, early clinical trials of volociximab demonstrated acceptable feasibility but only modest and context‐dependent antitumour activity. These findings suggest that α5β1‐targeted therapy may be insufficient in unselected patients and may require rational combination strategies and biomarker‐guided patient selection.

#### 6.1.2 PF‐04605412

PF‐04605412 represents a next‐generation therapeutic antibody with dual functional properties. This fully human IgG1 monoclonal antibody was designed not only to block the interaction between integrin α5β1 and FN, but also to elicit antibody‐dependent cellular cytotoxicity (ADCC). Preclinical studies demonstrated that PF‐04605412 effectively binds integrin α5β1 on tumour cells and triggers immune‐mediated cytotoxicity, thereby enhancing antitumour activity beyond simple integrin blockade.^121^ Subsequent early‐phase clinical studies evaluated the safety, pharmacokinetics and preliminary efficacy of PF‐04605412 in patients with advanced solid tumours. In a first‐in‐human phase I clinical trial, PF‐04605412 demonstrated evidence of target engagement and acceptable pharmacokinetic properties. However, infusion‐related reactions were observed, and the overall antitumour activity was limited, which ultimately restricted further clinical development of the antibody.^122^ PF‐04605412 thus illustrates the evolution of integrin‐targeted therapies from simple ligand‐blocking agents to multifunctional antibodies capable of engaging both tumour cells and the immune system.

### 6.2 Peptide antagonists

#### 6.2.1 ATN‐161

In addition to monoclonal antibodies, peptide antagonists targeting integrin α5β1 have also been developed to disrupt integrin‒ECM interactions. One of the most extensively studied compounds is ATN‐161 (Ac‐PHSCN‐NH_2_), a small peptide derived from the synergy region of FN that acts as a non‐RGD inhibitor of integrin α5β1. By interfering with the interaction between α5β1 integrin and FN, ATN‐161 disrupts integrin‐mediated adhesion and downstream signalling pathways that regulate tumour angiogenesis, migration, metastasis and tumour‐associated inflammation.^123^, ^124^

Preclinical studies have demonstrated that ATN‐161 exerts significant antitumour activity across several experimental models. In breast cancer xenograft models, ATN‐161 markedly inhibited tumour growth and metastatic dissemination by suppressing integrin‐dependent signalling and tumour angiogenesis.^125^ Similarly, in colorectal cancer liver metastasis models, inhibition of integrin α5β1 using ATN‐161 significantly reduced tumour burden and microvessel density, while improving survival when combined with continuous 5‐fluorouracil treatment. These findings highlight the potential of ATN‐161 not only as a direct inhibitor of tumour progression but also as a chemosensitising agent capable of enhancing the efficacy of conventional chemotherapy.^126^ Early clinical investigations further assessed the safety and pharmacological properties of ATN‐161. A phase I clinical trial in patients with advanced solid tumours demonstrated that ATN‐161 was well tolerated and exhibited a favourable safety profile, with no dose‐limiting toxicities observed. These results support the feasibility of further clinical development of integrin α5β1 antagonists and suggest that ATN‐161 may represent a promising candidate for integrin‐targeted cancer therapy.^127^

#### 6.2.2 AV3 and engineered cyclic AV3 derivatives

AV3 is an ITGA5‐targeting peptidomimetic supported by PDAC‐specific preclinical evidence. Kuninty et al. demonstrated that pharmacological inhibition of ITGA5 with AV3 in PSCs attenuated stromal activation, reduced desmoplasia, improved tumour perfusion, and potentiated the efficacy of gemcitabine in 3D heterospheroid, co‐injection and patient‐derived xenograft (PDX) models.^51^ More recently, cyAV3.3, an engineered cyclic AV3 derivative with high tumour accumulation and retention, was shown to disrupt PSC/CAF‐mediated interactions between tumour cells and the stroma, reduce ECM deposition and enhance gemcitabine sensitivity in PDAC models.^128^ These findings suggest that AV3‐derived peptides may represent a PDAC‐relevant strategy for stromal reprogramming and chemosensitisation, although they remain at the preclinical stage and require further validation before clinical translation.

### 6.3 Other experimental integrin α5β1 inhibitors

Small‐molecule antagonists such as SJ749 disrupt the interaction between integrin α5β1 and FN, thereby inhibiting integrin‐mediated adhesion and reducing tumour cell proliferation and clonogenic potential in experimental models.^129^ Notably, SJ749 has also been reported to modulate tumour cell responses to chemotherapy. In human glioblastoma cells, inhibition of α5β1 signalling reduces chemotherapy‐induced premature senescence and promotes apoptotic cell death, suggesting that α5β1 blockade may enhance therapeutic efficacy by shifting tumour cells from a senescent to an apoptotic phenotype.^110^

JSM6427 is a potent, selective small‐molecule antagonist of integrin α5β1 that has been extensively evaluated in preclinical models for its impact on tumour‐associated vascular and glioma growth. In models of hypoxia‐induced neovascularisation, JSM6427 inhibited endothelial cell migration and tube formation in vitro and significantly reduced pathological blood vessel formation in vivo, indicating that α5β1 signalling contributes to angiogenesis under oxygen‐deprived conditions and that its blockade can suppress aberrant vascular growth.^130^ Notably, in an experimental mouse glioma model, treatment with JSM6427 for 14 days following intracerebral implantation of glioma cells resulted in a significant reduction in tumour volume compared with vehicle controls. Flow cytometric analysis confirmed the expression of α5β1 on both glioma cells and microglia, and depletion of microglia abrogated the inhibitory effect of JSM6427 on glioma invasion, suggesting that integrin α5β1‐mediated interactions between tumour cells and stromal microglia contribute to glioma progression. Furthermore, JSM6427 attenuated microglial migration and proliferation in a dose‐dependent manner, highlighting its ability to modulate the glioma microenvironment in addition to direct effects on tumour cell dynamics.^131^ Together, these findings support the therapeutic potential of targeting α5β1 integrin with small‐molecule antagonists such as JSM6427 to disrupt both neovascularisation and tumour‒stroma crosstalk in solid tumours.

More recently, bispecific antibodies targeting both α5β1 and αv integrins have emerged as a novel strategy to overcome functional redundancy within the integrin family. A bispecific antibody directed against α5β1 and αv integrins has been shown to induce integrin internalisation and degradation in prostate cancer cells, leading to sustained suppression of integrin‐mediated signalling.^132^ Compared with monospecific antibodies, this dual‐targeting approach exhibited superior inhibitory effects on tumour cell adhesion, migration and survival. Importantly, this antibody also engages the immune system, promoting NK cell‐mediated tumour elimination, thereby integrating direct tumour inhibition with immune‐mediated cytotoxicity.^133^ These findings suggest that bispecific α5β1/αv integrin antibodies not only provide more effective blockade of integrin signalling than monospecific agents but also offer mechanistic innovations, including transcriptional reprogramming and immune activation, underscoring their potential as a next‐generation therapeutic strategy that combines integrin targeting with modulation of the tumour immune microenvironment.

### 6.4 Gene therapy approaches

#### 6.4.1 Plasmid AMEP

Beyond the therapeutic approaches described above, gene therapy strategies targeting integrin α5β1 have also been investigated. Plasmid AMEP represents a non‐viral gene delivery system encoding an integrin‐binding protein capable of targeting both α5β1 and αvβ3 integrins. Preclinical studies demonstrated that gene electrotransfer of plasmid AMEP exerts significant antitumour and antiangiogenic effects in murine B16 melanoma models, including inhibition of tumour growth and suppression of tumour‐associated vascularisation.^134^ Building on these preclinical results, a phase I clinical trial (NCT01664273) evaluated intramuscular gene electrotransfer of plasmid AMEP in patients with advanced solid tumours. The treatment was well tolerated and demonstrated a favourable safety profile, supporting the feasibility of this approach. Although the primary endpoint was safety, the study also assessed pharmacokinetics and preliminary efficacy, highlighting the translational potential of integrin‐targeted gene therapy.^135^ Taken together, these studies suggest that plasmid‐based delivery of integrin inhibitors via gene electrotransfer represents a promising alternative to conventional integrin‐targeted therapies, with the advantage of sustained protein expression and potential systemic effects.

### 6.5 Limitations and challenges of α5β1‐targeted therapy

Although integrin α5β1 is strongly implicated in tumour progression and stromal remodelling, its therapeutic targeting has thus far yielded limited clinical benefit. A major obstacle is the context‐dependent and functionally redundant nature of integrin signalling, which allows tumour cells and stromal elements to compensate for α5β1 inhibition through alternative adhesion pathways. In addition, experience from the broader integrin inhibitor field has shown that some antagonists may induce paradoxical receptor activation or proangiogenic effects at low concentrations, emphasising the need for structurally optimised compounds that maintain pure antagonism.^136^ Furthermore, integrin α5β1 functions primarily as a signalling modulator rather than a dominant oncogenic driver, which may partly explain the limited efficacy of monotherapy observed in clinical studies.

The modest clinical activity observed in early trials suggests that α5β1‐targeted therapy may be insufficient in unselected patients. Future strategies should therefore prioritise rational combinations with chemotherapy, stromal remodelling approaches or inhibitors of compensatory adhesion and survival pathways. In PDAC, such combinations may help alleviate the fibrotic stromal barrier, improve drug delivery and suppress tumour‒stroma‐mediated survival signalling. Multi‐target approaches, including bispecific antibodies targeting multiple integrins and gene‐based strategies such as plasmid AMEP, may also help overcome pathway redundancy and improve therapeutic durability. Biomarker‐guided patient selection is another important requirement for the clinical translation of α5β1‐targeted therapy. Potential biomarkers include high ITGA5 or FN1 expression, CAF/PSC‐associated stromal signatures, hypoxia‐related ECM remodelling signatures, and desmoplastic tumour features, which may help identify tumours more dependent on the FN‒α5β1 axis. However, these biomarkers remain to be prospectively validated in PDAC clinical trials.

## 7 CONCLUSIONS AND FUTURE PERSPECTIVES

PDAC is among the most lethal malignancies, largely due to its aggressive biology, profound therapeutic resistance, and a dense desmoplastic TME. Integrin α5β1, the principal receptor for FN, functions as an important mediator of ECM remodelling and therapeutic resistance by regulating cell‒matrix adhesion, mechanotransduction, stromal activation and downstream survival signalling. Within the hypoxic PDAC microenvironment, hypoxia‐driven activation of the FN‒α5β1 axis in PSCs/CAFs and tumour cells may promote ECM deposition, matrix stiffening and impaired drug delivery, thereby reinforcing treatment resistance. Early‐phase clinical trials have evaluated α5β1‐targeted strategies across several malignancies; although these approaches have generally demonstrated acceptable safety and feasibility, their clinical efficacy has remained modest.

Overall, integrin α5β1 represents a biologically relevant therapeutic target for modulating the fibrotic stroma and overcoming treatment resistance in PDAC, but successful clinical translation requires overcoming pathway redundancy, TME‐driven heterogeneity, and the limited efficacy of monotherapy. Future research should prioritise mechanistic studies, multi‐target or combination therapies and biomarker‐guided patient selection to fully exploit the therapeutic potential of α5β1 inhibition in PDAC and other solid tumours.

## AUTHOR CONTRIBUTIONS

Chenzhe Ma drafted the manuscript and contributed to visualisation and data curation. Yingying Wang contributed to data curation. Yumin Li supervised the study and contributed to the conceptualisation of the manuscript. All the authors have reviewed and approved the final manuscript.

## CONFLICT OF INTEREST STATEMENT

The authors declare they have no conflicts of interest.

## ETHICS STATEMENT

Not applicable.

## Data Availability

Data sharing does not apply to this article as no datasets were generated or analysed during the current study.
